# Yazidi Women: Healing the Invisible Wounds

**DOI:** 10.9745/GHSP-D-18-00024

**Published:** 2018-03-21

**Authors:** Dilshad Jaff

**Affiliations:** a Gillings School of Global Public Health, University of North Carolina at Chapel Hill, Chapel Hill, NC, USA.

## Abstract

Global health cannot be improved without addressing the plight of the survivors and victims of brutal armed conflicts, especially minorities and marginalized people.

While serving in hospitals and health facilities as a physician during the U.S. war in Iraq between 2003 and 2011, I witnessed firsthand the suffering of many vulnerable people and came face-to-face with the terrible acts of barbarity that we humans perpetrate against each other. I also discovered that every survivor has a unique and horrifying story to tell.

Among the greatest atrocities in the Middle East today are those committed against the Yazidis, a Kurdish-speaking religious and ethnic minority descended from the ancient peoples of Mesopotamia. The Yazidis, who number approximately 700,000, observe an ancient religion with elements of Zoroastrianism, Mithraism, and Islam.

Accused of being “devil worshipers,” the Yazidis have been persecuted for centuries. In 2014, ISIS massacred approximately 2,400 Yazidis; more than 600 were children and elderly adults. ISIS fighters also enslaved, tortured, and raped tens of thousands of women and children. During military operations, more than 3,000 people, mostly women and girls, were rescued. Some were released after paying ransom to ISIS fighters; some managed to escape their captors.

At present, more than 200,000 Yazidis have been displaced, and thousands of women and girls are still missing. Yazidi women have suffered the greatest physical and psychological consequences from the attacks by ISIS. Suicide, poverty, separation, and stigma shadow the lives of the survivors who live in pain and isolation. Their unseen psychological scars and poor physical health often inhibit their ability to reconnect with their families and Kurdish and Yazidi communities. While there are no accurate figures, it is estimated that there are very high rates of suicide, burning/self-immolation, and attempted suicide among the Yazidi survivors.

The condition in which Yazidi women find themselves is a major public health crisis that challenges the capabilities and resources of local authorities and the international community. To date, programs and interventions that could help these women are limited in scope and employ passive approaches that are short-term and small scale and that do not include local inputs. The volatile situation in Iraq and throughout the Middle East requires an ambitious vision; a clear and adaptable road map; and practical, tailored programs that allow survivors of atrocities to recover in safe and secure societies.

Without policies to promote inclusion, members of the Yazidi community and others will continue to feel isolated and in despair. Implementing effective policies and strategies calls for recognizing the devastating, long-term effects of the atrocities on the survivors and their communities, and it requires international organizations and civil society to implement effective resiliency policies and programs to provide care to these neglected survivors.

At present, there is no agency or program in place to address the needs of the Yazidi women. Agencies with the expertise and capacity to address these conditions should coordinate their efforts with others to introduce measures using community-based and participatory approaches.

To address the crisis, at least 3 steps must be taken.

- First, survivors should be guaranteed personal safety and security.
- Second, effective counseling is needed to allow the survivors to tell their stories—a measure that will help them realize that they are not alone.
- Third, support should be offered to empower each woman to see herself as worthy of respect and valued by her family and community. Each woman needs some psychosocial and material assistance, i.e., safe and secure housing, job skills, and employment.

Effective, coordinated support should be provided to the survivors to ensure that the Yazidi community has a stable and secure future. Global health cannot be advanced without addressing the plight of the victims and survivors of these brutal armed conflicts. Practitioners of global health must address this crisis along with other humanitarian challenges.

## Notes

**Funding:** None.

